# Bilateral Implantation of Shear Stress Modifier in *ApoE* Knockout Mouse Induces Cognitive Impairment and Tau Abnormalities

**DOI:** 10.3389/fnagi.2018.00303

**Published:** 2018-10-04

**Authors:** Shuke Nie, Yang Tan, Zhentao Zhang, Guiqin Chen, Jing Xiong, Dan Hu, Keqiang Ye, Yunjian Zhang, Xuebing Cao, Liam Chen, Zhaohui Zhang

**Affiliations:** ^1^Department of Neurology, Renmin Hospital of Wuhan University, Wuhan, China; ^2^Department of Pathology, Johns Hopkins University School of Medicine, Baltimore, MD, United States; ^3^Department of Neurology, Union Hospital, Tongji Medical College, Huazhong University of Science and Technology, Wuhan, China; ^4^Department of Pathology and Laboratory Medicine, Emory University School of Medicine, Atlanta, GA, United States

**Keywords:** vascular cognitive impairment, shear stress modifier, atherosclerosis, tau, asparagine endopeptidase

## Abstract

Vascular cognitive impairment (VCI) encompasses all causes of cerebrovascular disease that lead to cognitive decline, or overt dementia, atherosclerotic disease being the most common contributor. However, few rodent models that mimic the pathology of VCI replicated the clinical cerebrovascular atherosclerosis. Here we aimed to investigate the mechanism underlying VCI in an *Apolipoprotein E* knockout (*ApoE*-KO) mouse model fed with western style food with implantation of bilateral shear stress modifiers. We established a cognitive decline in spatial learning and memory developed in the bilateral modifier treated mice. Brain imaging and pathological examinations demonstrated reduced glucose intake and neuronal loss in hippocampus. Although no amyloid plaques or neurofibrillary tangles (NFTs) were observed, tau pathology including hyperphosphorylation, paired helical filament formation and pathologic truncation were found at considerable higher extent in the bilateral modifier group 8 weeks post the procedure. In addition, gliosis and microglia activation were confirmed in corpus callosum (CC) and ventral striatum. Thus, this *ApoE*-KO mouse model faithfully replicates the stenosis of common carotid artery (CCA) and cognitive impairment following atherosclerotic deposition and global cerebral hypoperfusion. The close correlation of cognitive decline and tau pathology indicates the toxic tau species could be at least partially responsible for the neurodegenerative changes induced by the chronic hypoxia/ischemia.

## Introduction

Vascular cognitive impairment (VCI) encompasses all causes of cerebrovascular disease that lead to cognitive decline, from mild cognitive impairment (MCI) to dementia (Iadecola, 2013). Second to Alzheimer’s disease (AD), VCI has emerged as a major cause of age-related dementia (Gorelick et al., 2011). Among various cerebrovascular diseases, atherosclerosis of carotid artery or cerebral blood vessels is the major contributor to VCI (Iadecola, 2013; Jellinger, 2013). Previous studies have found that atherosclerosis leads to hemodynamic change and narrowing of carotid artery, resulting in chronic cerebral hypoperfusion. Considering the fundamental role of the cerebral blood supply for the structural and functional integrity of the brain, it is not surprising that alterations in vascular structure and function have a profound impact on cognitive function (Ruitenberg et al., 2005; Balestrini et al., 2013; Iadecola, 2013). Indeed, cerebral hypoperfusion has been associated with cognitive impairment even without obvious ischemic lesions detected by magnetic resonance imaging (MRI) imaging (Johnston et al., 2004; Mathiesen et al., 2004; Balestrini et al., 2013). Nevertheless, the underlying mechanism connecting cerebral hypoperfusion and cognitive impairment is still elusive.

Several rodent models have been established in an attempt to address the question, including permanent bilateral occlusion of the common carotid arteries (2-vessel occlusion, 2-VO) in rats (Farkas et al., 2007), bilateral common carotid artery stenosis (BCAS; Shibata et al., 2004, 2007) and gradual common carotid artery stenosis (GCAS) in mice (Hattori et al., 2015). Being used as the most promising model of cerebral hypoperfusion, BCAS exhibits white matter rarefaction, gliosis and cognitive impairment (Shibata et al., 2004). However, it is difficult to determine whether the pathological changes were related to acute cerebral blood flow (CBF) drops or the short-term cerebral hypoperfusion because blood flow gradually recovered over 1 month after surgery (Ihara and Tomimoto, 2011). On the other hand, GCAS model exhibits chronic cerebral hypoperfusion, but the mechanical constriction failed to reliably replicate the atherosclerotic pathology (Hattori et al., 2015).

Here, we exploit a recently developed mouse model using a modifying cuff to induce disturbing blood flow in carotid artery in *Apolipoprotein E* knockout (*ApoE*-KO) mice fed with high cholesterol western type diet (Kuhlmann et al., 2012). Bilateral implantation of the shear stress modifier (BSSM) effectively accelerates atherosclerosis compared with mouse on normal diet and replicates chronic cerebral hypoperfusion (Kuhlmann et al., 2012), therefore providing an alternative platform to investigate the possible mechanism underlying chronic cerebral hypoperfusion and cognitive impairment.

## Materials and Methods

### Animal

Five-week-old male *ApoE*-KO mice and western type diet (H10141) were purchased from HFK Bioscience Company (Beijing, China). Mice were housed in barrier system under regulated temperature (21–23°C) with 12 h light/dark cycle with food and water available. This study was carried out in accordance with the principles of the Basel Declaration and recommendations of the National Institutes of Health guide for the care and use of laboratory animals (NIH Publications No.8023, revised 1978). The protocol was approved by the Institutional Animal Care and Use Committee (IACUC) at Tongji Medical College, Huazhong University of Science and Technology (HUST), China, with good laboratory practice and standard operating procedure.

### Antibodies and Materials

Akt (pan, cat#4691), phospho-Akt (Ser473, cat#4060), phospho-GSK3β (Ser9, cat#5558) were all rabbit antibodies and purchased from Cell Signaling (Danvers, MA, USA). Anti-GSK3β rat (cat#MAB2506), Legumain/asparaginyl endopeptidase sheep (Cat#AF2058) antibodies were purchased from R&D System (Minneapolis, MN, USA). Anti-GFAP (cat#ab7260), phosphor-Tau (S396, cat#ab109390) and PHF1 (cat#ab66275) rabbit antibodies were purchased from Abcam (Cambridge, UK). Anti-NeuN antibody (cat#SIG-39860) was obtained from Biolegend, San Diego, CA, USA. Anti-IAB1 antibody (cat#PA5–21274), anti-tau AT8 antibody (cat#MN1020) and DAPI (cat#62248) were obtained from Thermo Fisher Scientific, Waltham, MA, USA. All the secondary antibodies and anti-β-actin antibody were bought from Antegene (Wuhan, China). The thermoplastic polyetherketone cast elements, which were used as shear stress modifier, were purchased from Promolding (Den Haag, Netherlands). Anti-Tau, AEP-cleaved (N368) rabbit antibody was from Dr. Keqiang Ye at Emory University.

### Bilateral or Unilateral Implantation of a Shear Stress Modifier in Mice

We followed the shear stress-modifying cuff procedure that has been established by Schafers group (Kuhlmann et al., 2012). All mice were fed with western type diet for 4 weeks before implantation of casts. Mice were randomly divided into three groups: sham-operated, unilateral implantation of shear stress modifier (USSM) in which the modifier was implanted only around the left common carotid artery (CCA) and BSSM group. Each group contained 12 mice. Briefly, the half shells of a cast element with a 300 μm inner diameter at downstream end were cut off from the cast. Once the neck of the mouse was opened and CCA was exposed, two half shells were placed around the CCA and the half shells were tightened using a suture loop to form a cone shaped inner lumen.

### The Morris Water Maze Test

Morris water maze (MWM) tests were carried out 2 months after surgery to evaluate cognitive performance of each group as previously described (Gao et al., 2016). Briefly, A 120 cm diameter tank was filled with water with non-toxic milk powder dissolved in it. The pool was divided equally into four quadrants: northeast (NE), southeast (SE), southwest (SW) and northwest (NW). The circular platform was 10 cm in diameter located in the center of NE quadrant, and was submerged 1.5 cm below the surface of water. Several visual cues were placed in the room as spatial reference for mice to locate the invisible platform, and experimenter remained stationary in a fixed location. After 5 days of acquisition trials, the platform was removed and mice were placed in the SW quadrant, opposite to the former platform position and the probe trial underwent. During the whole trials, all mice were maintained on their western type diet. Behavioral parameters (latency; percentage of time and distance in the NE quadrant; the number of times the mouse crossing over the position of the platform) were recorded and evaluated with AVTAS software.

### Positron Emission Tomography-Computed Tomography (PET-CT) and Magnetic Resonance Imaging (MRI) Study

Three mice of each group were chosen randomly 2 months after surgery to undergo PET-CT and MRI examination. ^18^F-FDG was injected intravenously through tail vein (35 MBq in 0.2 ml normal saline). One hour after the injection, brain radioactivity concentration was acquired for 30 min (six frames of 5 min each). ^18^F-FDG uptake in the brain was expressed as mean standardized uptake value (SUV) using the following formula: (mean radioactivity measured in the volume of interest/injected radioactivity) * body weight. MRI (Bruker Biospec, performed at Chinese Academy of Science, Institute of Wuhan Physics and Mathematics) was used to detect any structure changes and lesion sites of cortex and hippocampus in the three groups. Representative slices of T2-weighted (T2WI) and diffusion weighted (DWI) image were chosen.

### Blood Management and Biochemical Analysis

Blood samples were obtained from the angular vein after MWM. Serum was separated by centrifuge and stored at −80°C until analysis. Serum levels of triglyceride (TG), cholesterol (CHO) and low-density lipoprotein (LDL) were determined by enzymatic methods using commercial kits (Jiancheng Bioengineering Institute, Nanjing, China).

### Tissue Preparation

After MWM tests, six mice of each group were sacrificed under anesthesia with 1% sodium pentobarbital. Brain tissues were dissected quickly on the ice and stored at −80°C after being rapidly frozen with liquid nitrogen. Four mice from each group were fixed with 4% paraformaldehyde solution by transcardially perfusion and the intact brains were taken and bathed in paraformaldehyde for 24 h before being embedded in paraffin. The entire aorta along with the heart was dissected under microscope.

### Oil Red O Staining

The arterial tree was washed in distilled water and immersed in 75% ethyl alcohol for 10 s. The artery was subsequently immersed in the oil red O solution (Goodbio, Wuhan, China) for 10 min before transferred to 75% ethyl alcohol for 8 s and washed with distilled water.

### Immunohistochemical Staining

Immunohistochemical staining was performed with primary antibodies recognizing NeuN (1:800), tau AT8 (1:800), GFAP (1:500) and IAB1 (1:1,000). Antigen retrieval was performed using a microwave for 3 min in 10 mM citrate buffer at pH 6.0. To calculate the number of NeuN positive neurons in hippocampus, three representative high-power field views from each of the CA1, CA2, CA3 and CA4 region were selected and only cells with unambiguously neuronal morphology were counted. Same areas were selected for phosphorylated tau AT8 staining to show the abnormal granular tau accumulation in the neuronal cell body. To investigate microglia activation and gliosis, three representative high-power field views from each of the corpus callosum (CC) and striatum area of individual mouse were selected and only cells with unambiguously IBA1 staining and highly ramified morphology were counted as activated microglia. Similarly, only cells with unambiguously GFAP positivity with either protoplasmic or fibrous astrocytic morphology were counted for gliosis.

### Immunofluorescent Staining

Formalin-fixed paraffin embedded tissue sections were deparaffinized in xylene, rehydrated by immersion in a descending series of ethanols. Antigen retrieval was performed using a microwave for 6 min in 10 mM citrate buffer solution (pH 6.0), followed by incubation in 0.3% H_2_O_2_ for 30 min. Tissues were blocked with 3% normal goat serum plus 0.3% Triton X-100 overnight. Primary anti-Tau N368 (1:500) was added and incubated for overnight at 4°C, subsequently incubated for 1 h with secondary Alexa Fluor 594 goat-anti rabbit (1:200) and counterstained with DAPI. Sections were mounted and cover slipped with anti-fade Vector mounting Medium. Images of hippocampal CA1 regions in each group were captured under inversion fluorescence microscope (NIKON DS-U3) and the optical density was evaluated by ImageJ software as described before (Nie et al., 2015).

### Silver Staining

After deparaffinization and washing with distilled water, slides were placed in pre-warmed (37°C) 10% silver nitrate solution for 20 min followed by distilled water wash for three times. Slides were then placed in ammonium silver solution and stained in 40°C oven for 25 min followed by immersion in developer working solution for 1 min. Slides were placed in 1% ammonium hydroxide solution for 1 min to stop the silver reaction. Slides were then placed in 5% sodium thiosulfate solution for 4 min, followed by distilled water wash, dehydrate and clear through 95% ethyl alcohol, absolute alcohol and xylene before mounting with resinous medium. Neurofibrillary tangles (NFTs) and amyloid plaques were stained black with a tan background.

### Western Blot Analysis

Protein preparation was carried out as previously described (Nie et al., 2015). A total of 42 μg proteins of each sample were separated by electrophoresis on 10% separating gels. After SDS-PAGE, the proteins were transferred onto PVDF membrane (Millipore, Bedford, MA, USA). The membrane was blocked for 2 h with 5% non-fat milk at room temperature followed by incubation with primary antibody: anti-Tau N368 (1:2,000); anti-Akt (pan; 1:1,000), anti-phospho-Akt Ser473 (1:2,000), anti-phospho-GSK-3β Ser9 (1:1,000), anti-phosphor-Tau S396 (1:4,000), anti-PHF1 (1:1,000), anti-β-actin (1:3,000); anti-GSK-3β (1:2,000); anti-Legumain/asparaginyl endopeptidase (1:8,000) at 4°C overnight. Blots were then washed and incubated with respective secondary antibody (1:2,000) for 2 h at 4°C. The protein expression was detected by a Bio-Rad imaging system, subsequently calculated by the ImageJ software.

### Statistical Analysis

All data were presented as mean ± standard deviation (SD). Data were analyzed using SPSS Statistics 22.0. Indexes in acquisition trails in the MWM were analyzed using a two-way repeated measures analysis of variance (ANOVA; treatment × day). One-way ANOVA was conducted on the data obtained from the probe trial in MWM and all other multiple group comparisons, followed by the least significant difference (LSD) *post hoc* comparisons.

## Results

### BSSM Caused Rapid Atherosclerotic Deposition in *ApoE* Knockout Mouse on High Fat Diet

**Figure 1:**
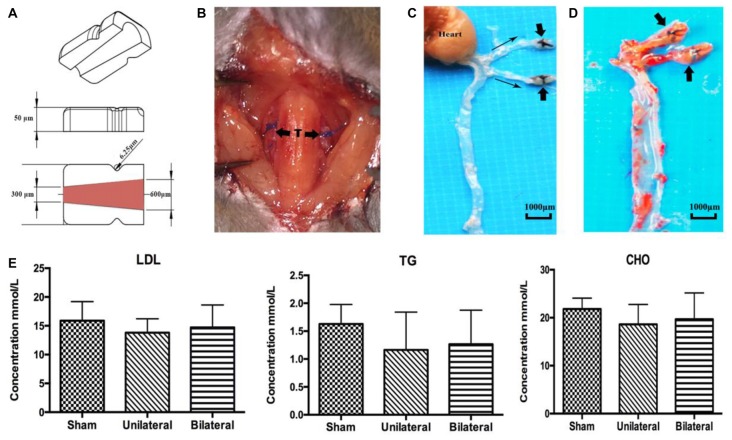
Shear stress modifier induces rapid atherosclerosis in mice. **(A)** Diagram of the shear stress modifier. The cuff imposes a gradual stenosis from 600 μm to 300 μm of inner diameter at downstream end with a cone shaped lumen. **(B)** BSSMs on the common carotid arteries. T, trachea; thick arrow, shear stress modifier. **(C,D)** Gross specimen of main arteries 8 weeks after bilateral placement of the shear stress modifier on high calorie diet. The opaque white regions of the vessels correspond to the sites of extensive atherosclerotic plaque deposition both upstream and downstream (thin arrow indicating the blood flow direction) of the cuff (thick arrow, **C**), better viewed after oil red O staining **(D)**. **(E)** No significant difference of the serum concentration levels of low-density lipoprotein (LDL), triglyceride (TG) and cholesterol (CHO) among sham (*n* = 12), USSM (*n* = 11) and BSSM (*n* = 9) groups. Sham: sham surgery group; Unilateral: unilateral implantation of shear stress modifier (USSM); Bilateral: bilateral implantation of shear stress modifier group (BSSM).

As expected, *ApoE*-KO mouse under high fat (western type) diet 8 weeks after unilateral or bilateral placement of the shear stress modifier around the CCA developed atherosclerotic lesions both upstream and downstream from the cuff (Figures 1A–D). In contrast, there were no signs of plaque deposition in the sham group or the contralateral side of the USSM group. However, there were no significant differences between each group on the serum concentration levels of TG, CHO and LDL (Figure 1E), indicating the rapid plaque deposition in these transgenic hyperlipidemic mouse models is induced by altered flow dynamics on the basis of high calorie diet.

### BSSM Reduced Glucose Intake and Increased Neuronal Loss in Hippocampus

**Figure 2:**
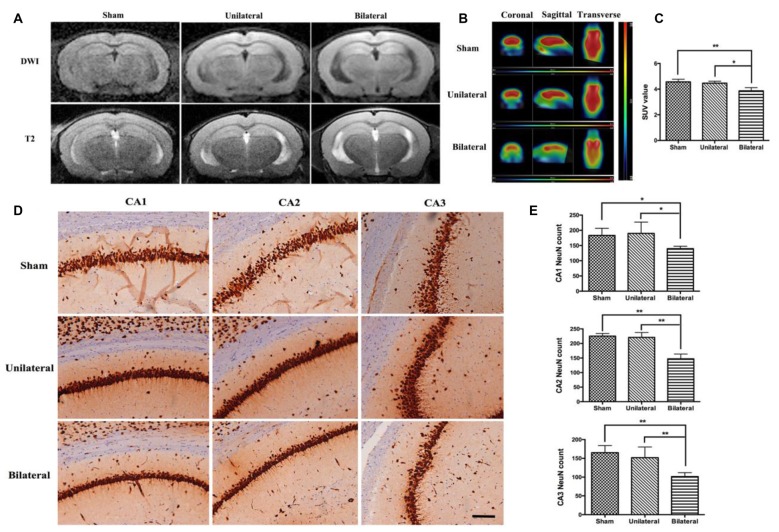
Decreased brain metabolism and hippocampal neuronal loss in BSSM group. **(A)** No obvious infarction was seen in each group on diffusion weighted imaging (DWI) or T2-weighted (T_2_WI) images (*n* = 2 in each group). **(B,C)** Decreased ^18^F-FDG uptake measured as mean standardized uptake value (SUV) in the BSSM group compared with the other two groups (*n* = 3 in each group). **(D,E)** Neuronal cell counts in the hippocampal CA1, CA2 and CA3 regions. Neurons were highlighted by NeuN immunohistochemical staining. Values were presented as Mean ± SD. *p < 0.05, **P < 0.01. Scale bar, 150 μm. Sham: sham group; Unilateral: unilateral implantation of shear stress modifier (USSM); Bilateral: bilateral implantation of shear stress modifier group (BSSM).

Next, we investigated possible parenchymal alterations associated with the vascular lesions. At 8 weeks after cuff implantation, no acute, subacute or chronic infarcts and hemorrhages were found by DWI or T2 sequence on MRI studies (Figure 2A). To investigate whether there are any brain metabolic changes associated with atherosclerosis, we performed PET-CT scan. We found that the glucose ^18^F-FDG uptake was significantly decreased in hippocampus in the BSSM group compared with the other two groups (*F*_(2,6)_ = 9.923, *P* = 0.0125, Figures 2B,C), which prompted us to examine closely whether there is associated cell loss in hippocampus. Indeed, NeuN staining and a careful cell count revealed that neuronal loss was occurring in CA1-CA3 regions of hippocampus in the BSSM group (*F*_(2,9)CA1_ = 5.450, *P* = 0.0281; *F*_(2,9)CA2_ = 36.49, *P* < 0.001; *F*_(2,9)CA3_ = 11.80, *P* = 0.0031; Figures 2D,E), compared with sham and USSM groups, whereas neurons in CA4 region is relatively spared (data not shown). Taken together, a combination of hypometabolic status and loss of neurons vulnerable to hypoxic-ischemic insult have been induced by the bilateral placement of the shear stress modifiers around the common carotid arteries.

### BSSM Was Associated With Impairment of Spatial Learning and Memory

**Figure 3:**
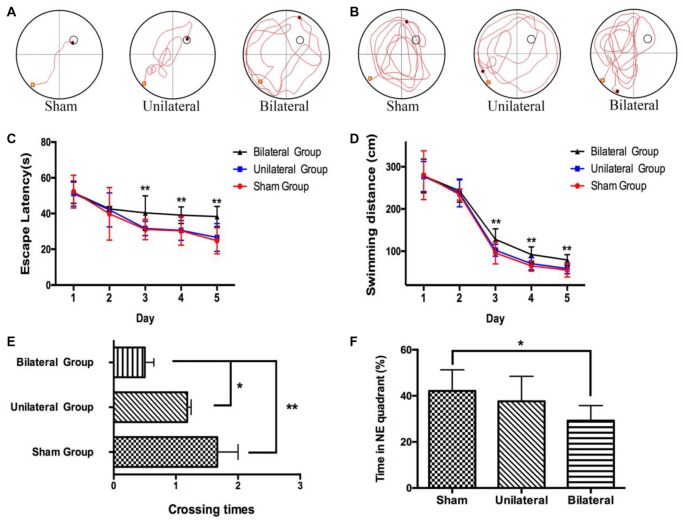
Impairment of spatial learning and memory in BSSM group. The swimming trajectory of each group on the last day of training trails **(A)** and probe trail **(B)**. The statistical analysis of escape latencies **(C)** and swimming distances **(D)** in 5 days training trails. The number of times that the mouse crossed over the place where the submerged platform was located **(E)** and the percentage of time in the target northeast (NE) quadrant **(F)** were analyzed in the probe trail. Sham (*n* = 9): sham group; Unilateral (*n* = 11): unilateral implantation of shear stress modifier (USSM); Bilateral (*n* = 12): bilateral implantation of shear stress modifier group (BSSM). Values were presented as Mean ± SD. *p < 0.05, **P < 0.01.

Meanwhile we performed MWM test, which is commonly thought to be related with hippocampal-dependent spatial learning and memory (D’Hooge and De Deyn, 2001), to assess the effects of cuff implantation on cognitive function of the animals. The swimming trajectories of mouse from each group on the last day of training trials (Figure 3A) and probe trials (Figure 3B) were shown. Measured by two-way ANOVA (day*treatment), the escape latencies (Day: *F*_(4,29)_ = 26.825, *P* < 0.0; Group: *F*_(2,29)_ = 3.736, *P* = 0.036; Interaction: *F*_(8,116)_ = 0.746, *P* = 0.651) and swimming distances (Day: *F*_(4,29)_ = 433.728, *P* < 0.01; Group: *F*_(2,29)_ = 6.849, *P* = 0.004; Interaction: *F*_(8,116)_ = 0.862, *P* = 0.551) during training trails showed remarkable differences between BSSM and the other groups. Compared with sham and USSM groups, BSSM group performed poorly on training days except for the first 2 days (Figures 3C,D). No significant difference was found between sham and USSM group in escaping from the pool. Similarly, we found that both the sham and USSM groups crossed over the zone where the hidden platform was located more frequently than the BSSM group in probe trial testing (Figures 3E, *F*_(2,29)_ = 7.171, *P* < 0.05). The swimming time spent in the target NE quadrant, the location of the hidden platform, was shorter for the BSSM mice, while longer for both sham and USSM groups (Figure 3F). Collectively, BSSM treatment leads to cognitive impairment of spatial learning and memory in *ApoE*-KO mice with western type diet.

### BSSM Promoted Tau Hyperphosphorylation Without Increased Amyloid Deposition or Neurofibrillary Tangle Formation in Hippocampus

**Figure 4:**
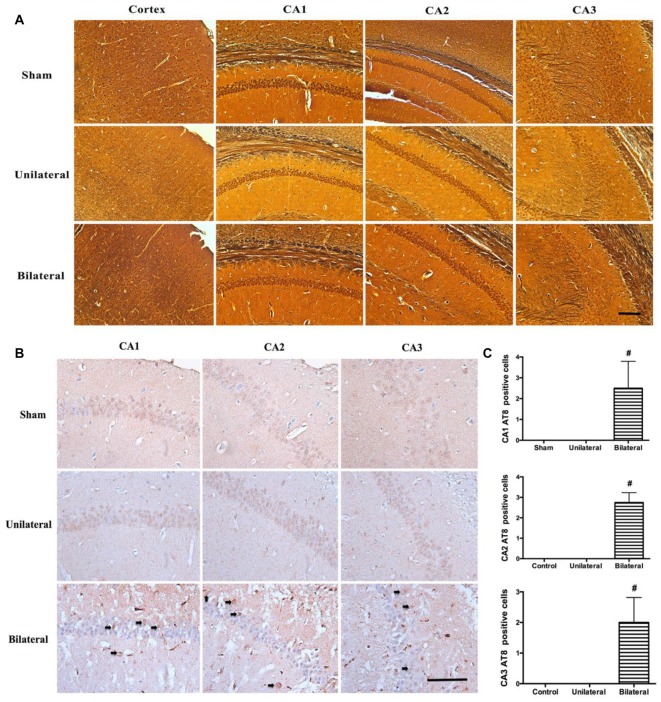
BSSM promotes tau hyperphosphorylation without amyloid plaque or neurofibrillary tangle (NFT) formation. **(A)** Silver staining of cortex and hippocampus. Scale bar, 200 μm; *n* = 3 in each group. **(B,C)** Phosphorylated tau (AT8) immnostaining in the CA1, CA2 and CA3 regions of hippocampus. Arrows show the granular, phosphorylated abnormal tau accumulation. Scale bar, 50 μm. Data were presented as Mean ± SD. ^#^*p* < 0.01. Sham: sham group; Unilateral: unilateral implantation of shear stress modifier (USSM); Bilateral: bilateral implantation of shear stress modifier group (BSSM).

Increased beta-amyloid deposition and pathologic tau hyperphosphorylation and NFT formation are hallmarks of AD and correlates tightly with the loss of neurons, compromised synapse integrity and function in medial temporal lobe and hippocampus (Augustinack et al., 2002). Therefore, we examined whether there is increased beta-amyloid accumulation and NFT formation by both modified Bielschowsky silver staining and immunohistochemical staining with antibodies against beta-amyloid and phosphorylated tau at pS202/pT205 (AT8). AT8 antibody was chosen because pS202 and pT205 are well presented in the NFT and phosphorylation at these sites on endogenous tau drops to a low level at age of 3 months in rodents and remain unchanged for the rest of life (Yu et al., 2009). No diffuse or neuritic plaques were seen in hippocampus on silver stains from all three groups (Figure 4A), nor were there any increased beta-amyloid deposition on immunohistochemical stains (data not shown). Similarly, no NFT formation can be seen in hippocampus on silver stains from all three groups (Figure 4A). Nevertheless, prominently increased abnormal hyperphosphorylated tau were detected as granular deposition in hippocampal CA1, CA2 and CA3 neurons in BSSM group compared with sham and USSM groups (*F*_(2,7)CA1_ = 10.50, *P* = 0.0078; *F*_(2,7)CA2_ = 23.10, *P* = 0.0008; *F*_(2,7)CA3_ = 10.81, *P* = 0.0072; Figures 4B,C). Taken together, BSSM treatment specifically targets tau protein and enhances its conversion to a well-known hyperphosphorylated, pathologic precursor preceding NFT formation.

### BSSM Activated AKT/GSK3β Pathway, Enhanced Asparagine Endopeptidase and Promoted Pathologic Tau Conversion

**Figure 5:**
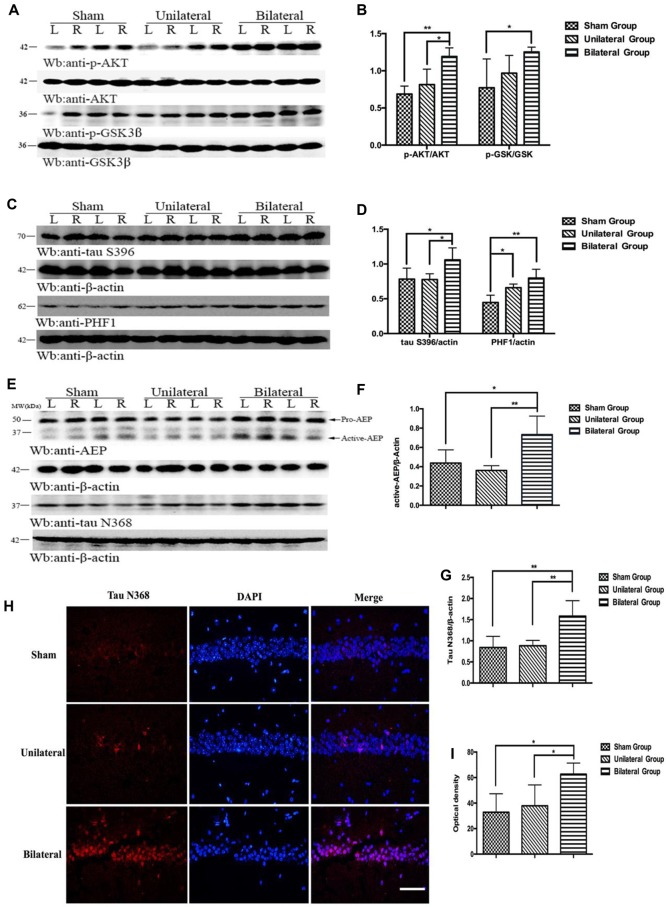
BSSM activates AKT/GSK3β pathway, promotes tau hyperphosphorylation and PHF accumulation and increases the expression of active asparagine endopeptidase (AEP) and tau cleavage. **(A,B)** Western blotting showed higher expressions of p-AKT and the downstream p-GSK in hippocampus in BSSM treated group. **(C,D)** Phosphorylation of tau at S396 was higher in BSSM group than in sham and USSM group. PHF accumulation also showed significant differences between sham and the two shear stress modifier groups. **(E–G)** Expression of active AEP and tau N368 truncation were higher in BSSM group. **(H,I)** Immunofluorescent staining further reconfirmed the finding that BSSM group had more tau N368 fragments in hippocampus than the other two groups. L, left hippocampus; R, right hippocampus. *p < 0.05, **p < 0.01. Data were presented as Mean ± SD. *N* = 4 in each group. Scale bar, 100 μm.

Previous study has shown that the phosphoinositide 3-kinase (PI3K)/protein kinase B (also known as AKT) signal pathway is activated after 2-VO treatment and there is a linear relationship between the PI3K protein expression and the behavioral measures of the cognitive abilities (Shu et al., 2013), strongly implicating a neuroprotective effect following chronic cerebral hypoperfusion. PI3K/AKT pathway is known to inhibit the kinase activity of glycogen synthase kinase-3β (GSK-3β) through phosphorylation of Ser9 (Jiang et al., 2015). We assessed the activation of PI3K/AKT pathway in mice hippocampus with western blotting (Figures 5A,B). Indeed, BSSM treatment dramatically up-regulated the expressions of AKT phosphorylation (p-AKT) compared with those in USSM (*p* < 0.05) and sham-operated group (*p* < 0.01). Difference was also found between sham group and BSSM-treated group on expression of GSK-3β phosphorylation (p-GSK-3β, *p* < 0.05). Nevertheless, the poor performance of BSSM group on spatial learning and memory suggests that the neuroprotective effects associated with PI3K/AKT activation is not sufficient to compensate the detrimental effects of chronic cerebral hypoperfusion. GSK-3β is one of the major tau kinases, especially at S396 and S404 sites (Liu et al., 2007). As tau hyperphosphorylation could lead to pathologic accumulation of paired helical filaments (PHF; Sun and Chen, 2015), we further evaluated the alteration of tau phosphorylation at S396 and abnormal conformation conversion in hippocampus. We found that phosphorylated-tau at serine 396 was dramatically increased in BSSM group compared with other groups (*p* < 0.05, Figures 5C,D) despite the decreased GSK-3β activity, strongly arguing that BSSM treatment also increases the enzymatic activities of serine/threonine protein kinases other than GSK-3β. Not surprisingly, there were also significant differences on PHF1 levels between sham and BSSM group (*p* < 0.01; Figures 5C,D). Interestingly, although there was no apparent difference on tau S396 phosphorylation between USSM and sham group, USSM treatment did promote more PHF accumulation, indicating phosphorylation at sites other than S396 may be responsible for the pathologic tau conversion.

Besides phosphorylated tau, truncated tau also was found to play an important role in tau-related neurodegeneration (Zhang et al., 2014). Asparagine endopeptidase (AEP), a lysosome cysteine proteinase that is synthesized as a zymogen (pro-AEP, 56 kDa) and autocatalytically processed into active AEP (36 kDa) under acidic conditions following deficiency of CBF (Basurto-Islas et al., 2013), could proteolytically cleave tau at N368 site, eliciting insoluble fibril aggregation and neurofibrillary degeneration (Zhang et al., 2014). To investigate whether levels of active AEP would increase in BSSM treated *ApoE*-KO mice with severe atherosclerosis, we evaluated the expression of AEP and the alteration of tau N368 truncated by active-AEP. Indeed, we found significant higher active-AEP expression in BSSM group (Figures 5E,F) than sham-operated group (*p* < 0.05) and USSM group (*p* < 0.01). Correspondingly, the amount of tau N368 fragments was higher in BSSM group compared with sham-operated and USSM group (both *p* < 0.01; Figures 5E,G). The level of tau N368 in the hippocampus was also examined by immunofluorescence staining. Tau N368 in hippocampus of BSSM group was significantly increased compared with the other two groups (both *p* < 0.05; Figures 5H,I). Taken together, the cognitive impairment of *ApoE*-KO mice is tightly associated with increased tau pathology including hyperphosphorylation, paired helical filament formation and cleavage by increased active-AEP in hippocampus.

### BSSM Induced Microglia and Astrocytic Activation in the Subcortical White Matter

**Figure 6:**
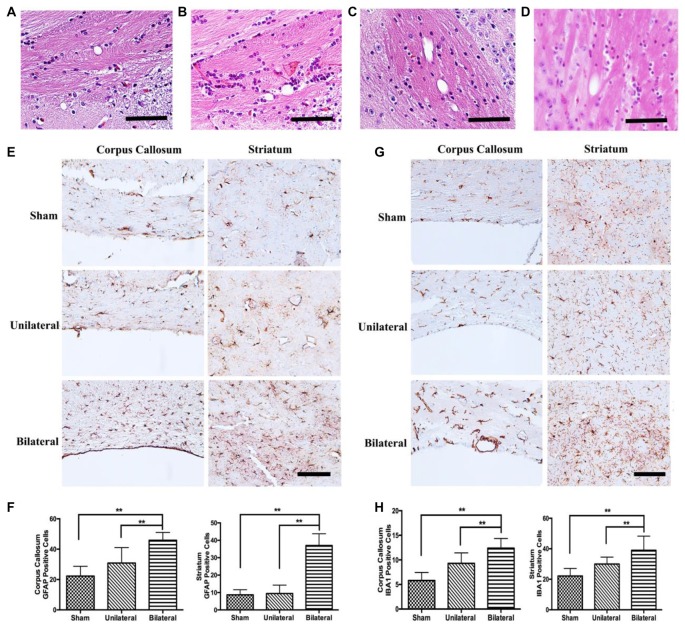
Gliosis and microglial activation in BSSM group. **(A–D)** Vacuole formation in corpus callosum (CC) and the fiber bundles of the caudoputamen were found in mice 8 weeks post BSSM treatment. Scale bar, 50 μm. **(E,F)** The proliferation of astrocytes in the CC and striatum were significantly higher in the BSSM group compared with Sham and USSM groups. Data were presented as Mean ± SD. **p < 0.01. Scale bar, 150 μm, *n* = 3 in each group. **(G,H)** IAB1 staining in the CC and ventral striatum of the three groups. Data were presented as Mean ± SD. **p < 0.01. Scale bar, 150 μm, *n* = 3 in each group. Sham: sham group; Unilateral: unilateral implantation of shear stress modifier (USSM); Bilateral: bilateral implantation of shear stress modifier group (BSSM).

Cerebrovascular white matter lesions are closely associated with cognitive impairment and are believed to be caused by chronic cerebral hypoperfusion (Shibata et al., 2004). The white matter including the CC was consistently found to be rarefied in BCAS model (Shibata et al., 2007; Nishio et al., 2010). Interestingly, rarefaction and vacuole formation in CC and caudoputamen were a consistent feature in mice 8 weeks post BSSM treatment (Figures 6A–D), indicating BSSM can induce white matter lesion as well. To further confirm the white matter injury and associated glia activation, we performed GFAP and IAB1 staining in CC and ventral striatum. As expected, the proliferation of astrocytes in the CC and striatum were significantly higher in the BSSM group compared with Sham and USSM groups (*F*_(2,18)cc_ = 17.43, *P* < 0.001; *F*_(2,18)striatum_ = 17.43, *P* < 0.001), indicating a robust astrocytic response after hypoperfusion (Figures 6E,F). Importantly, microglia activation in CC and striatum of BSSM group is much more prominent than those in USSM and sham groups (*F*_(2,19)cc_ = 19.51, *P* < 0.001; *F*_(2,15)striatum_ = 9.782, *P* = 0.0019; Figures 6G,H), consistent with the observation that disturbed blood flow with low and reciprocating shear stress caused by the cast and the vascular stenosis can rapidly set in motion an inflammatory cascade (Chiu and Chien, 2011). Together, microglia activation and gliosis are accompanying the tissue rarefaction and vacuolization in the white matter, strongly supporting that BSSM can induce subcortical white matter injury.

## Discussion

Cerebrovascular disease, either large vessel disease or chronic hypoperfusion manifesting as lacunae and white matter disease, is a major contributor to cognitive impairment and dementia (Jellinger, 2013; Horsburgh et al., 2018). There is an unmet need to provide animal models and mechanistic insights to improve therapeutic interventions. Here we for the first time, demonstrate a particular impairment in hippocampus-dependent spatial learning and memory in *ApoE*-KO mice received bilateral implantation of shear stress cuffs, which is supported by the findings of a hypometabolic state and hypoxic-ischemic sensitive neuronal loss in the CA1–3 regions of the hippocampus. In addition, various manifestations of tau pathology and evidence of subcortical white matter injury are presented. Thus, long-term hypoperfusion in the BSSM model induces a pronounced cognitive impairment coincident with pathological alterations and more accurately replicates features of human VCI.

Compared to other well-developed rodent models of cerebral hypoperfusion such as 2-VO, BCAS or GCAS, our study established a causal relationship of chronic cerebral hypoperfusion and cognitive impairment while offering two unique advantages. First, it faithfully replicate the atherosclerotic lesions. Other group has proved that prominent atherosclerosis could be developed in as early as 6 weeks with shear stress modifier (Cheng et al., 2006). Although the mechanisms by which different risk factors such as hyperlipidemia, hypertension and diabetes may impact VCI are still poorly understood, considerable evidence has demonstrated that endothelial dysfunction is pivotal to the pathophysiology (Di Marco et al., 2015). Therefore, the BSSM model is an ideal *in vivo* model to investigate the consequences of endothelial dysfunction and inflammatory responses induced by shear stress implantation and altered flow dynamics, conditions most reminiscent of human vascular diseases. Second, BSSM implantation drastically accelerates the cognitive decline. In our previous study, *ApoE*-KO mice fed western type diet functioned normally in the behavioral test until 30 weeks old (Tan et al., 2016). Similarly, both spatial working memory and spatial reference memory were impaired in BCAS model only after long-term, i.e., 6 months of hypoperfusion (Nishio et al., 2010). Intriguingly, the USSM group did not show spatial memory deficits by 8 weeks. We chose to implant the cuff around left CCA based on the study that cognitive decline is associated with asymptomatic high-grade stenosis of the left internal carotid artery (Johnston et al., 2004). We suppose that collateral circulation from the right side might help ameliorate the hypoperfusion in USSM group. Alternatively, it may take longer than 8 weeks to observe any significant deficits.

Subcortical vascular dementia is primarily caused by small vessel disease involving white matter changes. Nevertheless, white matter lesion are observed in rodent models of chronic cerebral hypoperfusion (Shibata et al., 2004, 2007) and here in our study as well. Spatial reference memory task is related to cognitive domains that mainly rely on the integrity of the hippocampus, thus impaired reference memory is in agreement with the neuronal loss in the hippocampus. In contrast, working memory impairment may be attributable to either frontal white matter lesions or hippocampal damages (Shibata et al., 2007). It would be very interesting and potentially significant to examine whether white matter changes and working memory impairment present at an early stage post BSSM treatment. The CBF of the white matter is lower than that of the gray matter (Tsuchiya et al., 1992), might induce different histological changes under the hypoperfusion condition. An inflammatory process in the central nervous system by microglial activation is believed to play an important role in the pathway leading to neuronal cell death (Dheen et al., 2007). It has been demonstrated that perivascular shear stress modifier could induce regions of lowered/oscillatory shear stresses and release proatherogenic inflammatory mediators to prompt the plaque formation of carotid arteries (Cheng et al., 2006). It is interesting to find that the microglia activation in the CC and ventral striatum of the BSSM group is much more prominent than sham group, supporting the glia-related neuro-inflammation in VCI.

Although the contribution of cerebral large vessel disease to multi-infarct vascular dementia has been widely studied, the mechanism by which chronic hypoperfusion contributes to dementia, particularly AD dementia, remains poorly understood. Longitudinal studies on cognitively normal elderly subjects have demonstrated that the impact of amyloid and vascular pathologies are at least partly independent processes and additive (Vemuri et al., 2015), suggesting cerebral hypoperfusion may be working via non-amyloid pathways to promote the neurodegeneration. It is worth pointing out that no formation of amyloid plaque was found in the cortex and hippocampus regions in our study. Besides Aβ amyloid, abnormal hyperphosphorylation of tau and pathologic conversion to PHF conformation and NFTs are a key driver to neuronal death in AD and other tauopathies (Sun and Chen, 2015). Here we demonstrated in BSSM group a cohort of abnormal tau pathology exist in the absence of definitive NFTs, including phosphorylation at S396 and S202/T205(AT8), increased PHF formation and cleaved tau N368 fragments by enhanced active-AEP activity. It is known that synapse loss is an early event in the AD disease process (Scheff et al., 2006), and patient with AD suffer from early synaptic dysfunction prior to tau aggregate formation (Zhou et al., 2017). Thus, the hippocampal-dependent spatial learning and memory impairment is most likely due to a combined effect of tauopathy and loss of hippocampal neurons that are more sensitive to hypoxic-ischemic insults. Interestingly, this happens in the context of PI3K/AKT pathway activation and subsequent down-regulation of GSK3b kinase activity, whose major phosphorylation sites are S396 and S404. However, this result is not surprising given the human brain utilizes almost 50% of the total body glucose under the basal condition (Peters, 2011) and that decreased brain glucose metabolism could regulates tau phosphorylation inversely through down-regulation of tau O-GlcNAcylation (Li et al., 2006; Liu et al., 2009) at all the phosphorylation sites in hippocampus (Zhao et al., 2014). Indeed, a decreased glucose intake was observed in BSSM mice hippocampus. The neuronal loss and upregulated AEP activity seen in BSSM group are further evidence of a chronic hypoxia and acidic environment in the hippocampal area.

We acknowledge that lack of CBF measurement over the 8 weeks is a potential limiting factor to our study. Indeed, in a chronic cerebral hypoperfusion rat model caused by permanent bilateral carotid artery ligation (BCAL), local CBF was reduced to between 25–94% of the control at 2.5 h after BCAL but tended to recover 1 week after BCAL except for the regions of neuronal damage (Tsuchiya et al., 1992). On the other hand, previous studies have demonstrated that the mouse model used in our study develops conical stenosis of the vessel and subsequent atherosclerotic plaques, including inflamed, rupture-prone plagues upstream of the cuff and more stable plaques downstream of the cuff 8 weeks after cuff implantation (Wenning et al., 2014). It reliably elicits the atherosclerotic pathology, exhibits long-term cerebral hypoperfusion and therefore overcomes the inherent shortcomings of other alternative animal models.

In summary, we have established a causal role of chronic hypoperfusive state and cognitive impairment in an *ApoE*-KO mouse model that faithfully replicate the atherosclerotic vascular pathology. Our initial study demonstrates tau pathology and neurodegeneration in hippocampus, gliosis and microglia activation in the whiter matter, making this model a valuable *in vivo* platform to investigate various vascular risk factors and structural and functional changes that may lead to dementia.

## Author Contributions

SN, YT and Z-HZ conceived and designed the experiments, drafted the manuscript. YT, JX and DH performed the experiments and analyzed the data. LC conceived, drafted and revised the manuscript. GC, Z-TZ, XC, KY and YZ contributed reagents, materials and analysis tools. All authors read and approved the final manuscript.

## Conflict of Interest Statement

The authors declare that the research was conducted in the absence of any commercial or financial relationships that could be construed as a potential conflict of interest.
